# Proteinuria and Psoriasis Risk: A Nationwide Population-Based Study

**DOI:** 10.3390/jcm10112356

**Published:** 2021-05-27

**Authors:** Eun Hui Bae, Bongseong Kim, Su Hyun Song, Tae Ryom Oh, Sang Heon Suh, Hong Sang Choi, Chang Seong Kim, Seong Kwon Ma, Kyung-Do Han, Soo Wan Kim

**Affiliations:** 1 Department of Internal Medicine, Chonnam National University Medical School & Hospital, Gwangju 61469, Korea; baedak76@gmail.com (E.H.B.); sudang_@naver.com (S.H.S.); tryeomoh@hanmail.net (T.R.O.); medssh1984@gmail.com (S.H.S.); hongsang38@hanmail.net (H.S.C.); laminion@hanmail.net (C.S.K.); drmsk@hanmail.net (S.K.M.); 2 Department of Statistics and Actuarial Science, Soongsil University, Seoul 06978, Korea; qhdtjd12@gmail.com

**Keywords:** proteinuria, psoriasis, random urine

## Abstract

Psoriasis, a chronic inflammatory dermatosis, has been associated with chronic kidney disease or end-stage renal disease. However, the association of the changes or amount of proteinuria with psoriasis development has not been evaluated. Using the Korean National Health Screening database, we assessed psoriasis development until 2018 in 6,576,851 Koreans who underwent health examinations in 2009 and 2011. Psoriasis was defined using the International Classification of Diseases, 10th revision (ICD-10) code L40. The risk of psoriasis was evaluated according to change in proteinuria (never [Neg (no proteinuria)/Neg], new [Neg/Pos (proteinuria present)], past [Pos/Neg] and persistent [Pos/Pos] proteinuria) and the proteinuria amount. During a median 7.23-year follow-up, 162,468 (2.47%) individuals developed psoriasis. After adjustments, the hazard ratio (HR) for psoriasis was higher in the persistent proteinuria group (1.32 [1.24–1.40]) than in the never proteinuria group. The past proteinuria group showed better renal outcome (1.03 [1.00–1.07]) than the new (1.05 [1.01–1.07]) and never proteinuria (reference, 1.00) groups did. The amount of random urine proteinuria was associated with increased HR for psoriasis. Subgroup analyses for age, sex, estimated glomerular filtration rate (eGFR), hypertension and diabetes showed that the persistent proteinuria group had a higher risk of psoriasis than the never proteinuria group, especially at eGFR < 60 mL/min/1.73 m^2^. Persistent proteinuria is associated with psoriasis risk, and the proteinuria amount significantly affects psoriasis development.

## 1. Introduction

Psoriasis is a chronic inflammatory skin disease that affects 1–3% of the general population [1,2]. The prevalence rates vary between countries and ethnic groups. It is more common in Caucasians and in countries with higher latitudes and equally affects men and women [3]. The prevalence of psoriasis has been showing an increasing trend in certain countries [2,4,5]. The etiology of this disorder is not known; however, several risk factors have been identified, including smoking, obesity and alcohol abuse [6,7,8,9]. T helper (Th) 1 and Th17 lymphocytes are the major regulatory cells known to be involved in the pathogenesis of psoriasis [10]. Interestingly, studies have demonstrated that Th17 lymphocytes can induce inflammation in the kidneys by mediating tubular epithelial cells, mesangial cells and macrophages [11].

Inflammation plays a pivotal role in the pathogenesis of chronic kidney disease (CKD). Proteinuria is not only a marker but also a risk factor for CKD. A previous study has shown that proteinuria is significantly associated with psoriasis [12]. In addition, psoriasis increases the risk of CKD or end-stage renal disease [13,14]. However, whether proteinuria affects the development of psoriasis is unknown.

In this large population-based study, we aimed to investigate whether the changes in proteinuria and the amount of proteinuria are associated with a subsequent risk of psoriasis development. This study was conducted using data from the Korean National Health Insurance System (NHIS), which includes > 6.7 million Koreans with at least two proteinuria measurements.

## 2. Materials and Methods

Owing to the confidentiality of the data used in this study and the strict privacy policy of the data holder, the data were kept strictly among the designated research personnel and cannot be provided to others regardless of anonymity.

### 2.1. Study Design and Database

The Korean National Health Insurance Service manages a complete set of health information pertaining to 50 million Koreans and includes an eligibility database, a medical treatment database, a health examination database and a medical care institution database [15,16,17]. The National Health Insurance Corporation (NHIC), managed by the Korean government, is the sole insurer, to which approximately 97% of the Korean population are subscribed. Enrollees in the NHIC are recommended to undergo a standardized medical examination at least every 2 years.

**Figure 1:**
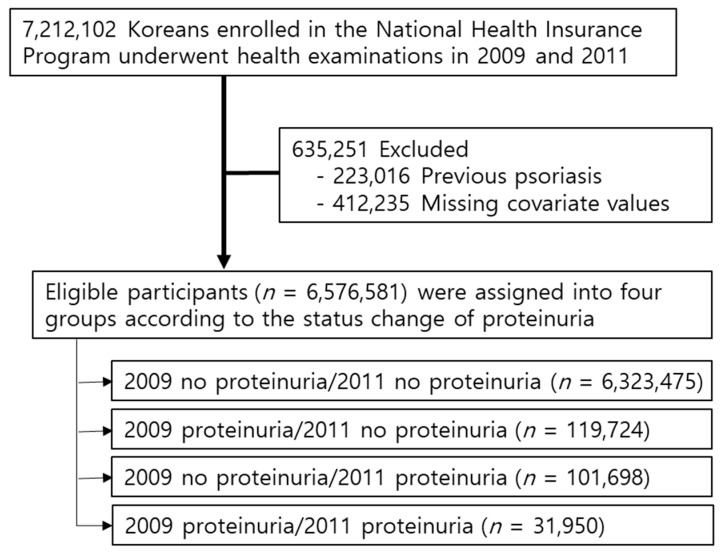
Study design.

According to the NHIS database, 7,212,102 Koreans underwent health examinations in 2009 and 2011 (index year). We excluded participants who had a previous diagnosis of psoriasis (Internal Classification of Disease, 10th revision (ICD-10) code L40., *n* = 223,016) [18] and those with missing information for at least one variable (*n* = 412,235). Ultimately, 6,576,581 participants were included in this study (Figure 1).

This study was approved by Chonnam National University Hospital (approval no. CNUH-EXP-2021-110) and the National Health Insurance Service and was conducted in accordance with the principles of the Declaration of Helsinki. The need for written informed consent was waived by our institutional review board.

### 2.2. Measurements and Definitions

The participants were tested for proteinuria using the dipstick method. The proteinuria status was defined as negative, trace and from 1+ to 4+. The never proteinuria (Neg [no proteinuria]/Neg) group included participants with no proteinuria both in 2009 and 2011. The persistent proteinuria (Pos [proteinuria present]/Pos) group comprised participants with proteinuria both in 2009 and 2011. The past proteinuria (Pos/Neg) group consisted of participants with proteinuria in 2009 and without proteinuria in 2011. The new proteinuria group (Neg/Pos) consisted of participants without proteinuria in 2009 but with proteinuria in 2011. Blood pressure (BP) was measured during the health examination by trained medical staff using auscultatory or oscillometric methods. The BP measurement protocol recommended at least 5 min of rest in a seated position followed by two repeated measurements with 5-min intervals [19]. Body mass index (BMI) was calculated as weight in kilograms divided by the square of height in meters. Information on current smoking and alcohol consumption status was obtained using a questionnaire. Heavy alcohol drinking was defined as an alcohol consumption of > 30 g/day. Regular exercise was defined as physical activity performed at least five times per week. Income level was dichotomized at the lowest 25%. Blood samples for the measurement of serum glucose and total cholesterol levels were drawn after an overnight fast. Comorbidities were identified using information collected in the 1 year before the index date. Hypertension was defined as a previous hypertension diagnosis (ICD-10 codes I10–13, I15) and a history of taking at least one antihypertensive drug or a recorded systolic BP ≥ 140 mmHg or diastolic BP ≥ 90 mmHg in the health examination database. Diabetes was identified using the appropriate diagnostic codes (E11–14) and a medical history of diabetes or a recorded fasting serum glucose level of ≥ 126 mg/dL in the health examination database. Dyslipidemia was identified using the appropriate diagnostic code (E78) and a history of use of lipid-lowering drugs or a total serum cholesterol level of ≥ 240 mg/dL in the health examination database. CKD was defined as an estimated glomerular filtration rate (eGFR) < 60 mL/min/1.73 m^2^ calculated using the CKD Epidemiology Collaboration equation. The quality of the laboratory tests has been validated by the Korean Association for Laboratory Medicine and the hospitals participating in the NHIS health examination programs are certified by the NHIS.

### 2.3. Study Outcomes and Follow-Up

The primary end point of the study was newly diagnosed psoriasis, which was defined as a new record of ICD-10 code L40. in the database for the registered participants during the follow-up period.

### 2.4. Statistical Analysis

Data are reported as mean ± standard deviation with intervals for continuous variables and as number (percentage) for categorical variables. To assess the risk of psoriasis related to proteinuria, we calculated the hazard ratios (HRs) with 95% confidence intervals (CIs) and analyzed these data using Cox proportional hazard regression models. We analyzed the associations between proteinuria and psoriasis development using three models: model 1, non-adjusted model; model 2, adjusted for age and sex; and model 3, adjusted for model 2 plus smoking, alcohol drinking, physical activity, BMI, low income, dyslipidemia, diabetes mellitus (DM) and eGFR. We also performed subgroup analysis for clinically important variables. A *p*-value < 0.05 was considered to indicate statistical significance. SAS version 9.4 software and SAS survey procedures (SAS Institute Inc., Cary, NC, USA) were used for all statistical analyses.

## 3. Results

### 3.1. Baseline Characteristics

Table 1 shows the baseline characteristics of the participants with respect to the development of psoriasis. Among all participants, 162,468 (2.47%) developed psoriasis. The mean age was higher in participants who developed psoriasis than in those who did not (48.83 ± 13.52 vs. 51.53 ± 13.82 years, *p* < 0.001). The proportions of participants with male sex (59.83%), obesity (BMI ≥ 25 kg/m^2^) and abdominal obesity (waist circumference ≥ 90 cm in men and ≥ 85 cm in women) were higher in the incident psoriasis group than in the non-psoriasis group. Comorbidities such as DM, hypertension and dyslipidemia were more prevalent in the psoriasis group than in the non-psoriasis group. eGFR was lower, whereas BP, total cholesterol and glucose levels were higher in the psoriasis group than in the non-psoriasis group (Table 1).

Of the participants, 6,323,475 (96.17%) were included in the never proteinuria group, 119,728 (1.82%) in the past proteinuria group, 101,695 (1.62%) in the new proteinuria group and 31,950 (0.48%) in the persistent proteinuria group. Compared to the other groups, the persistent proteinuria group was older and had more comorbidities such as DM, hypertension and dyslipidemia. The persistent proteinuria group also had higher BMI, waist circumference, fasting glucose levels, diabetes and dyslipidemia incidence and triglyceride levels; further, it included more male participants and current smokers than the never proteinuria group did (Table 2).

### 3.2. Effects of Changes in Proteinuria on Psoriasis Development

With the never proteinuria group as the reference, the multivariable adjusted HRs (95% Cis) for psoriasis outcome were 1.05 (1.01–1.09) for the past proteinuria group, 1.03 (1.00–1.07) for the new proteinuria group and 1.32 (1.24–1.40) for the persistent proteinuria group (Table 3). We also evaluated the effect of the amount of proteinuria on psoriasis development. According to the proteinuria amount, the HR for psoriasis was increased in both 2009 and 2011 (Table 4).

### 3.3. Subgroup Analyses

Subgroup analyses for CKD, which was defined as eGFR < 60 mL/min/1.73 m^2^, showed an increased psoriasis risk in the past, new and persistent proteinuria groups compared with that in the never proteinuria group. However, in the case of eGFR ≥ 60 mL/min/1.73 m^2^, only the persistent proteinuria group showed a higher HR for psoriasis. The HR for psoriasis increased according to the amount of proteinuria only for participants with eGFR < 60 mL/min/1.73 m^2^.

**Figure 2:**
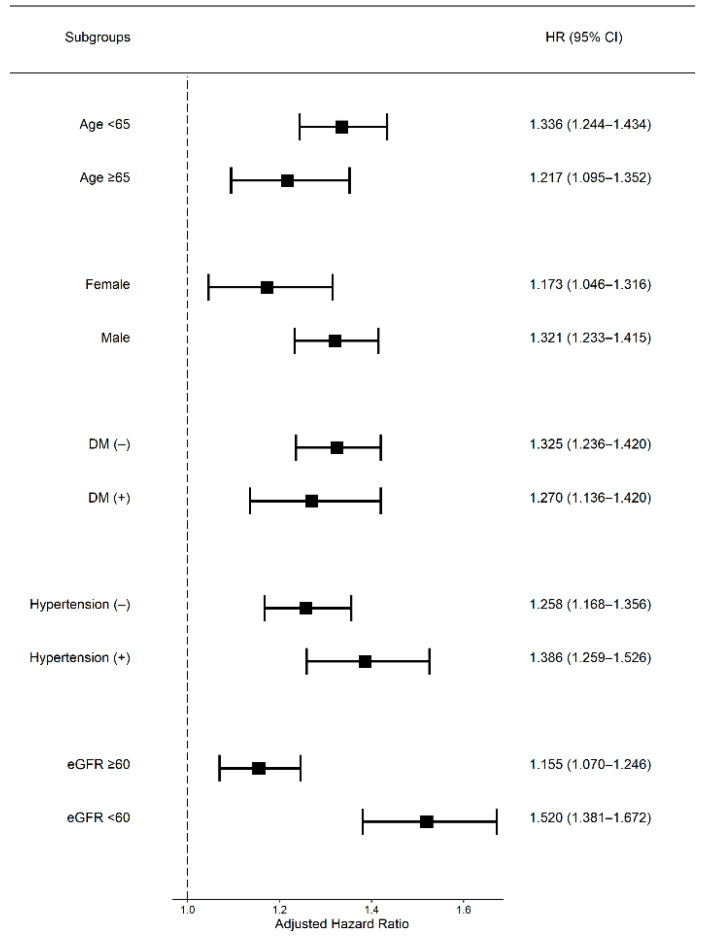
Forest plot of the subgroup analysis of risk of psoriasis in the persistent proteinuria group. Abbreviations: DM, diabetes mellitus; eGFR, estimated glomerular filtration rate; HR, hazard ratio; CI, confidence interval.

In the case of eGFR ≥ 60 mL/min/1.73 m^2^, only the 4+ proteinuria group showed a significantly increased risk of psoriasis (Table 5). In subgroup analyses for age, sex, DM and hypertension, the HR for psoriasis was not different between subgroups. Only the CKD group showed a significant difference between eGFR < 60 mL/min/1.73 m^2^ and eGFR ≥ 60 mL/min/1.73 m^2^ (Figure 2).

## 4. Discussion

In this large population-based study, we found that proteinuria was associated with the risk of psoriasis and the new proteinuria group had a higher risk than the past proteinuria group. We also observed that a greater proteinuria amount was associated with a greater risk of psoriasis development. Below 3+ proteinuria did not have an effect on psoriasis development at eGFR ≥ 60 mL/min/1.73 m^2^; however, even 1+ proteinuria significantly increased the risk of psoriasis at eGFR < 60 mL/min/1.73 m^2^. This association persisted after multivariable adjustment for possible confounding variables.

Previous studies focused on the prevalence of proteinuria in patients with psoriasis. The prevalence of proteinuria in patients with psoriasis has been reported to be 22–42% [20,21]. Many studies have reported controversial results about the association between proteinuria and psoriasis. Madeddu et al. [22] reported a high prevalence of proteinuria in patients with diffuse psoriasis; however, their study cohort included patients with arterial hypertension and DM. Cecchi et al. [20] demonstrated an increased prevalence of proteinuria in patients with psoriasis without other diseases that could be responsible for the development of proteinuria; however, Kaftan et al. [23] were unable to find any differences in the proteinuria prevalence between patients with psoriasis and healthy individuals.

The reasons for the increased prevalence of proteinuria in patients with psoriasis are not clear. A previous study has shown that in patients with atherosclerosis proteinuria correlated with circulating levels of soluble vascular cell adhesion molecule-1 (VCAM-1) [24]. Increased expression of VCAM-1 was also found in the skin and plasma of patients with psoriasis [25,26]. In addition, psoriasis is a Th1 and Th17 cell-mediated chronic inflammatory disease [10]. CKD can also induce Th17 lymphocytes as a result chronic inflammation in the kidneys [11].

As mentioned earlier, although a few studies have focused on the prevalence of proteinuria in patients with psoriasis, our study differs from these studies in that we investigated the occurrence of psoriasis in participants with or without proteinuria. The exact mechanism of how proteinuria affects psoriasis development is not fully understood. A possible explanation is that proteinuria is a biomarker and a risk factor for CKD, which is increased in inflammatory conditions such as psoriasis. Recently, emerging evidence has been reported that the role of gut microbiota, which closely interacts with the inflammation, kidney, cardiovascular and endocrine systems via metabolic, humoral and neural signaling pathways, causes chronic systemic inflammation, proteinuria, hypertension, diabetes and kidney disease [27]. Proteinuria could indicate the leakiness of glomerular capillaries as a consequence of damaged vascular endothelium [28]. Proteinuria is a predictor of the development of severe renal impairment, cardiovascular mortality and morbidity in patients with DM and arterial hypertension [28,29]. Generally, proteinuria is considered a subclinical marker of diffusely altered vascular permeability and endothelial damage in both diabetic and non-diabetic patients [28]. The increased incidence of essential hypertension and DM and enhanced plasma renin activity in patients with psoriasis is of special interest [30]. Moreover, it has been demonstrated that individuals with psoriasis have a higher incidence of peripheral vascular disease than those without psoriasis [31].

Our study showed that participants with persistent proteinuria had an obviously increased risk of psoriasis; however, new proteinuria development was also associated with a higher risk of psoriasis than that seen with past proteinuria occurrence. These results suggest the possibility that treatment of proteinuria using anti-proteinuric drugs such as renin-angiotensin-aldosterone system inhibitors might reduce psoriasis development. However, more systematic studies are needed to confirm this hypothesis.

### Limitations

This study had several limitations. First, the data source of this study was the NHIS database, which lacks relevant clinical variables such as laboratory data and pathologic findings. Second, co-medications are not coded in the database and anti-proteinuria drug-related effects could have an influence on our study results. Third, we did not consider the severity of psoriasis, but only assessed its incidence.

## 5. Conclusions

Persistent proteinuria is associated with psoriasis risk, and the proteinuria amount significantly affects psoriasis development.

## Figures and Tables

**Table 1:** Baseline characteristics of participants according to psoriasis incidence.

Group	Total (*n* = 6,576,851)	Non-Psoriasis Group (*n* = 6,414,383)	Psoriasis Group (*n* = 162,468)	*p* Value
Age (years)	48.89 ± 13.53	48.83 ± 13.52	51.53 ± 13.82	<0.0001
Sex, male (%)	3,747,448 (56.98)	3,650,244 (56.91)	97,204 (59.83)	<0.0001
Current smoking				
None	3,848,988 (58.52)	3,758,772 (58.6)	90,216 (55.53)	<0.0001
Ex	1,121,581 (17.05)	1,090,826 (17.01)	30,755 (18.93)	
Current	1,606,282 (24.42)	1,564,785 (24.39)	41,497 (25.54)	
Drinking				
None	3,337,444 (50.75)	3,252,694 (50.71)	84,750 (52.16)	<0.0001
Mild	2,764,312 (42.03)	2,698,978 (42.08)	65,334 (40.21)	
Heavy ^1^	475,095 (7.22)	462,711 (7.21)	12,384 (7.62)	
Physical activity, regular	1,340,250 (20.38)	1,306,224 (20.36)	34,026 (20.94)	<0.0001
Income, low ^2^	1,114,375 (16.94)	1,085,828 (16.93)	28,547 (17.57)	<0.0001
BMI (kg/m^2^)	23.83 ± 3.18	23.82 ± 3.18	24 ± 3.15	<0.0001
Waist circumference (cm)	80.62 ± 9.00	80.6 ± 9.00	81.62 ± 8.97	<0.0001
Diabetes mellitus	401,418 (6.10)	389,720 (6.08)	11,698 (7.20)	<0.0001
Hypertension	921,687 (14.01)	896,970 (13.98)	24,717 (15.21)	<0.0001
Dyslipidemia	718,083 (10.92)	699,779 (10.91)	18,304 (11.27)	<0.0001
Fasting blood glucose (mg/dL)	96.74 ± 22.39	96.71 ± 22.36	97.84 ± 23.65	<0.0001
Systolic blood pressure (mmHg)	122.35 ± 14.69	122.33 ± 14.69	122.92 ± 14.71	<0.0001
Diastolic blood pressure (mmHg)	76.36 ± 9.88	76.35 ± 9.88	76.55 ± 9.82	<0.0001
Total cholesterol (mg/dL)	195.43 ± 40.62	195.42 ± 40.62	195.96 ± 40.63	<0.0001
Estimated GFR (mL/min/1.73 m^2^)	87.21 ± 45.40	87.23 ± 45.46	86.37 ± 43.09	<0.0001
F/U duration (years)	7.23 ± 0.93	7.32 ± 0.67	3.75 ± 2.14	<0.0001

Data are presented as mean ± standard deviation or frequency (%). Abbreviations: CKD, chronic kidney disease; BMI, body mass index; GFR, glomerular filtration rate; F/U, follow-up. ^1^ Alcohol consumption ≥ 30 g/day. ^2^ Low income 25%.

**Table 2:** Baseline characteristics of the study population according to proteinuria status.

Characteristics	Never Proteinuria (Neg/Neg) (*n* = 6,323,475)	Past Proteinuria (Pos/Neg) (*n* = 119,728)	New Proteinuria (Neg/Pos) (*n* = 101,698)	Persistent Proteinuria (Pos/Pos) (*n* = 31,950)	*p* Value
Psoriasis event	155,413 (2.46)	3186 (2.66)	2744 (2.7)	1125 (3.52)	<0.0001
Follow-up duration (years)	7.24 ± 0.91	7.14 ± 1.12	7.08 ± 1.25	6.91 ± 1.48	<0.0001
Age (years)	48.73 ± 13.49	52.43 ± 13.90	52.77 ± 14.31	55.87 ± 13.15	<0.0001
Sex, male (%)	3,604,171 (57.00)	64,272 (53.68)	57,672 (56.71)	21,333 (66.77)	<0.0001
Income ^1^	1,068,434 (16.9)	22,119 (18.47)	18,210 (17.91)	5612 (17.56)	<0.0001
Exercise ^2^	1,287,705 (20.36)	24,902 (20.80)	20,912 (20.56)	6731 (21.07)	<0.0001
Smoking					<0.0001
None	3,699,938 (58.51)	72,828 (60.83)	59,386 (58.39)	16,836 (52.69)	
Ex-	1,075,202 (17.00)	20,709 (17.30)	18,423 (18.12)	7247 (22.68)	
Current	1,548,335 (24.49)	26,191 (21.88)	23,889 (23.49)	7867 (24.62)	
Drinking					<0.0001
None	3,199,180 (50.59)	66,013 (55.14)	54,373 (53.47)	17,878 (55.96)	
Mild	2,669,906 (42.22)	44,552 (37.21)	38,523 (37.88)	11,331 (35.46)	
Heavy	454,389 (7.19)	9163 (7.65)	8802 (8.66)	2741 (8.58)	
DM	359,676 (5.69)	15,426 (12.88)	17,737 (17.44)	8579 (26.85)	<0.0001
HTN	861,774 (13.63)	24,016 (20.06)	24,827 (24.41)	11,070 (34.65)	<0.0001
Dyslipidemia	684,227 (10.82)	14,033 (11.72)	14,490 (14.25)	5333 (16.69)	<0.0001
BMI (kg/m^2^)	23.8 ± 3.16	24.34 ± 3.47	24.39 ± 3.57	25.05 ± 3.65	<0.0001
WC (cm)	80.54 ± 8.96	82.1 ± 9.59	82.55 ± 9.79	85.1 ± 9.70	<0.0001
Glucose (mg/dL)	97.07 ± 21.44	104.1 ± 32.44	109.01 ± 37.87	118.63 ± 46.63	<0.0001
SBP (mmHg)	122.36 ± 14.48	125.21 ± 15.75	126.91 ± 17.01	132.09 ± 17.29	<0.0001
DBP (mmHg)	76.28 ± 9.75	77.49 ± 10.41	78.57 ± 11.01	80.71 ± 11.24	<0.0001
TC (mg/dL)	195.04 ± 36.48	194.55 ± 38.66	197.67 ± 40.82	198.9 ± 45.20	<0.0001
HDL	55.22 ± 21.92	54.2 ± 19.28	54.43 ± 34.85	51.61 ± 17.17	<0.0001
LDL	114.58 ± 44.17	112.92 ± 44.37	115.89 ± 56.72	114.64 ± 77.38	<0.0001
GFR (mL/min/1.73 m^2^)	89.6 ± 37.71	86.18 ± 39.33	83.98 ± 36.21	72.26 ± 42.26	<0.0001
TG	111.63 (111.58–111.68)	119.23 (118.84–119.62)	121.87 (121.43–122.32)	144.46 (143.54–145.38)	<0.0001

Data are presented as mean ± standard deviation or frequency (%). Abbreviations: Neg, no proteinuria; Pos, proteinuria present; SBP, systolic blood pressure; DBP, diastolic blood pressure; DM, diabetes mellitus; HTN, hypertension; CKD, chronic kidney disease; WC, waist circumference; BMI, body mass index; TC, total cholesterol; GFR, glomerular filtration rate; HDL, high-density lipoprotein; LDL, low-density lipoprotein; TG, triglyceride; AST, aspartate aminotransferase; ALT, alanine aminotransferase; rGTP, gamma glutamyl transferase. ^1^ Low income 25%. *p* value for all category < 0.0001. ^2^ Regular exercise: moderate exercise ≥5 days/week or vigorous exercise ≥3 days/week.

**Table 3:** Multivariable Cox analysis for incident psoriasis according to changes in proteinuria status.

Proteinuria	Total (*n*)	Psoriasis (*n*)	IR (per 1000)	HR (95% Confidence Interval)		
				Model 1	Model 2	Model 3
Never (Neg/Neg)	6,323,475	155,413	3.40	1 (ref.)	1 (ref.)	1 (ref.)
Past (Pos/Neg)	101,698	2744	3.81	1.12 (1.08–1.17)	1.06 (1.02–1.10)	1.05 (1.01–1.09)
New (Neg/Pos)	119,728	3186	3.73	1.10 (1.06–1.14)	1.04 (1.01–1.08)	1.03 (1.00–1.07)
Persistent (Pos/Pos)	31,950	1125	5.10	1.51 (1.42–1.60)	1.33 (1.26–1.41)	1.32 (1.24–1.40)
*p* value				<0.0001	<0.0001	<0.0001

Abbreviations: Neg, no proteinuria; Pos, proteinuria present; IR, incidence rate; HR, hazard ratio; ref., reference. Model 1: non-adjusted model. Model 2: adjusted for age and sex. Model 3: adjusted for model 2 plus low income, smoking, alcohol drinking, physical activity, body mass index, hypertension, dyslipidemia, diabetes mellitus and estimated glomerular filtration rate.

**Table 4:** Multivariable Cox analysis for incident psoriasis according to significance of proteinuria.

Proteinuria Significance	Total (*n*)	Psoriasis (*n*)	IR (per 1000)	HR (95% Confidence Interval)		
				Model 1	Model 2	Model 3
2011						
Negative	6,313,688	155,308	3.40	1 (ref.)	1 (ref.)	1 (ref.)
trace	129,515	3291	3.54	1.04 (1.01–1.08)	1.01 (0.98–1.05)	1.01 (0.98–1.05)
1+	88,245	2426	3.88	1.14 (1.10–1.19)	1.07 (1.03–1.11)	1.06 (1.02–1.11)
2+	34,340	1068	4.46	1.31 (1.24–1.40)	1.20 (1.13–1.27)	1.19 (1.12–1.26)
3+	9353	307	4.81	1.42 (1.27–1.59)	1.26 (1.13–1.41)	1.25 (1.12–1.40)
4+	1710	68	5.93	1.78 (1.40–2.25)	1.60 (1.27–2.03)	1.58 (1.25–2.00)
*p* trend				< 0.0001	< 0.0001	< 0.0001
2009						
Negative	6,279,838	154,350	3.40	1 (ref.)	1 (ref.)	1 (ref.)
trace	145,335	3807	3.64	1.07 (1.04–1.11)	1.04 (1.01–1.08)	1.04 (1.01–1.08)
1+	103,394	2802	3.80	1.12 (1.08–1.16)	1.06 (1.02–1.10)	1.05 (1.01–1.09)
2+	37,429	1115	4.23	1.25 (1.18–1.32)	1.15 (1.08–1.22)	1.14 (1.08–1.21)
3+	9107	333	5.29	1.57 (1.41–1.75)	1.42 (1.27–1.58)	1.40 (1.26–1.56)
4+	1748	61	5.08	1.50 (1.17–1.93)	1.34 (1.04–1.72)	1.33 (1.03–1.70)
*p* trend				< 0.0001	< 0.0001	< 0.0001

Abbreviations: IR, incidence rate; HR, hazard ratio; ref., reference. Model 1: non-adjusted model. Model 2: adjusted for age and sex. Model 3: adjusted for model 2 plus low income, smoking, alcohol drinking, physical activity, body mass index, hypertension, dyslipidemia, diabetes mellitus and estimated glomerular filtration rate.

**Table 5:** Multivariable Cox analysis for incident psoriasis according to eGFR.

Proteinuria	Total (*n*)	Psoriasis (*n*)	IR (per 1000)	HR (95% CI) Model 3	Proteinuria	Total (*n*)	Psoriasis (*n*)	IR (per 1000)	HR (95% CI) Model 3
eGFR < 60 mL/min/1.73 m^2^					eGFR ≥ 60 mL/min/1.73 m^2^				
Never	312,727	8848	3.96	1 (ref.)	Never	6,010,748	146,565	3.37	1 (ref.)
Past	12,695	427	4.88	1.13 (1.02–1.24)	Past	107,033	2759	3.59	1.02 (0.98–1.06)
New	13,018	474	5.40	1.24 (1.13–1.36)	New	88,680	2270	3.59	1.01 (0.97–1.05)
Persistent	10,075	454	6.86	1.52 (1.38–1.67)	Persistent	21,875	671	4.35	1.16 (1.07–1.25)
*p* value				< 0.0001	*p* value				0.0023
eGFR < 60 mL/min/1.73 m^2^					eGFR ≥ 60 mL/min/1.73 m^2^				
Negative	314,640	8906	3.96	1 (ref.)	Negative	5,999,048	146,402	3.37	1 (ref.)
trace	10,782	369	4.90	1.16 (1.05–1.29)	Trace	118,733	2922	3.42	0.99 (0.95–1.03)
1+	11,831	432	5.37	1.22 (1.11–1.35)	1+	76,414	1994	3.66	1.02 (0.98–1.07)
2+	7750	318	6.21	1.40 (1.25–1.57)	2+	26,590	750	3.98	1.09 (1.01–1.17)
3+	2923	148	7.95	1.76 (1.50–2.08)	3+	6430	159	3.52	0.94 (0.81–1.10)
4+	589	30	8.11	1.79 (1.25–2.56)	4+	1121	38	4.89	1.34 (0.98–1.84)
*p* trend				<0.0001	*p* trend				0.0817

Abbreviations: IR, incidence rate; HR, hazard ratio; Neg, no proteinuria; Pos, proteinuria present; eGFR, estimated glomerulus filtration rate; CI, confidence interval. Model 3: adjusted for age, sex, low income, smoking, alcohol drinking, physical activity, body mass index, hypertension, dyslipidemia, diabetes mellitus and estimated glomerular filtration rate.

## Data Availability

Data availability is bound by NHIS.
